# The practice of a modified bubble CPAP therapy in a rural Sierra Leone SCBU-A pilot study

**DOI:** 10.3389/fped.2025.1534550

**Published:** 2025-04-24

**Authors:** Fanghua Qin, Cuilan Dong, Jun Qiu, Qingqing Song, Kumba Konomanyi, Lucinda Sia Fatmata Sesay, Aiqing Xiao

**Affiliations:** ^1^ Department of NICU, The Affiliated Children’s Hospital of Xiangya School of Medicine, Central South University (Hunan Children’s Hospital), Changsha, Hunan, China; ^2^ Pediatrics Research Institute of Hunan Province, The Affiliated Children’s Hospital of Xiangya School of Medicine, Central South University (Hunan Children’s Hospital), Changsha, Hunan, China; ^3^ Department of Cardiology, The Affiliated Children’s Hospital of Xiangya School of Medicine, Central South University (Hunan Children’s Hospital), Changsha, Hunan, China; ^4^ SCBU, Sierra Leone–China Friendship Hospital, Freetown, Sierra Leone

**Keywords:** modified bubble CPAP, neonatal respiratory distress, respiratory support, resource-limited settings, clinical efficacy

## Abstract

**Background:**

Continuous Positive Airway Pressure (CPAP) is an effective intervention for managing neonatal respiratory distress. However, its implementation encounters numerous challenges in resource-limited settings. It is imperative for healthcare professionals to devise highly practical and cost-effective modifications to CPAP systems to address these challenges.

**Objective:**

To evaluate the clinical efficacy and operational feasibility of a modified bubble CPAP therapy utilizing locally available materials in reducing respiratory distress and improving survival rates of neonates in the Special Care Baby Unit (SCBU) of the Sierra Leone-China Friendship Hospital.

**Materials and methods:**

93 neonates with respiratory distress were divided into the control group (*n* = 48) for nasal cannula oxygen therapy and the observation group (*n* = 45) for modified bubble CPAP therapy. The modified CPAP device was constructed using locally available materials, such as drinking cups and modified nasal cannulas, with oxygen delivered via concentrators. Respiratory Severity Score, blood oxygen saturation, heart rate, and respiration were monitored with data recorded at admission and 8 h after intervention.

**Results:**

After intervention, the neonates in the observation group showed significant improvements in all parameters: decreased RSS scores (7.40 ± 0.986 vs. 5.33 ± 1.447, *P* < 0.001), heart rate (153.13 ± 5.998 vs. 141.60 ± 8.830, *P* < 0.001), and respiratory rate (47.87 ± 4.103 vs. 43.47 ± 3.833, *P* < 0.01), and higher oxygen saturation (73.60% ± 10.636% vs. 91.07% ± 8.940%, *P* < 0.001) and survival rate (88% vs. 62.5%, *P* < 0.01).

**Conclusion:**

The study indicated that the implementation of modified bubble CPAP therapy enhanced respiratory outcomes and increased survival rates among neonates experiencing respiratory distress in a resource-constrained setting in rural Sierra Leone.

## Introduction

Sierra Leone has one of the highest neonatal mortality rates (NMR) in the world, with 35 deaths per 1,000 live births. Approximately 21 babies die each day before reaching one month of age, compounded by 15 stillbirths daily (1). According to the World Health Organization (WHO), preterm birth and birth asphyxia remains a major cause of neonatal mortality in Sierra Leone (2) with respiratory distress syndrome (RDS) being a common complication of preterm birth that affects newborns' breathing. RDS, a leading cause of morbidity and mortality in low- and middle-income countries (LMICs) (3) arises from pulmonary immaturity and surfactant deficiency in newborns, leading to respiratory dysfunction shortly after birth.

While CPAP combined with pulmonary surfactant (PS) remains the gold standard for neonatal RDS management in most countries (4), its implementation still face challenges in hospitals with limited resources due to the cost and complexity of implementation. The WHO strongly recommends CPAP as the the treatment of premature infants with RDS (5), given its capability to enhance lung capacity, especially functional residual capacity. As increased positive airway dilation pressure can improve oxygenation, reduce apnea, and reduce work of breathing. CPAP has been successfully used to stabilize premature infants in both high- and low- to medium-income countries for over 50 years (6). It is recommended as the first choice for primary and secondary respiratory support in these infants (7, 8).

Despite the existence of costly CPAP devices in high-resource settings, CPAP can be applied with minimal technology or cost. A functional bubble CPAP can be implemented using three essential components: (1) an oxygen source to provide an appropriate concentration of oxygen, (2) a water bottle to generate positive pressure by submerging the distal end of the tubing in the water at the specific depth, and (3) a nasal cannula that connects the tubing to the baby's airway for pressurized air delivery. This simplified non-invasive ventilation method is known as bubble CPAP, which has been safely and effectively used to improve the survival of infants with various forms of neonatal respiratory distress in low- and middle-income countries (9). Yet, in low-income countries, the shortages of basic medical supplies often necessitate adaption and innovation with available resources from healthcare professionals. In Sierra Leone, for instance, researchers have creatively repurposed readily available drinking cups as water-seal bottles and customized existing nasal cannulas with scissors to create a modified bubble CPAP device.

The Sierra Leone-China Friendship Hospital, co-funded by the United Nations Children's Fund and the Chinese government, has a 10-bed special care baby unit (SCBU) that started modified bubble CPAP therapy since 2023, offering respiratory support to neonates with respiratory distress. This pilot study aims to evaluate the clinical efficacy and feasibility of modified bubble CPAP as primary respiratory support for neonates in the SCBU of Sierra Leone-China Friendship Hospital.

The study was approved by the Institutional Review Board of Hunan Children's Hospital, China, and the Sierra Leone-China Friendship Hospital (HCHLL2020-63). We report the study subjects' physiological response to therapy and the implementation outcomes of modified bubble CPAP. Written informed consent was obtained from the parents of all subjects after detailed disclosure of the study's purpose, procedures, and potential risks and benefits.

## Materials and methods

### Setting

This is a pilot study conducted at Sierra Leone—China Friendship Hospital, which is a regional referral medical center managing over 2,000 pediatric patients annually, serves as the sole provider of neonatal intensive care in the region. The SCBU is staffed by one assigned physician providing daily rounds for all neonates and 10 nurses (2–3 nurses per shift). In 2022, SCBU admitted 405 infants. During the 24-month study period, SCBU maintained a mean daily census of 10 neonates with a 2:1 patient-to-nurse ratio. The facility faced considerable resource constraints, including the lack of laboratory blood gas analyzer, x-ray machine and surfactant therapy during the study. Supplemental oxygen through nasal cannula and oxygen concentrators represented the sole respiratory support available for newborns with respiratory distress before this study.

### Patients

This study enrolled 93 neonates with respiratory distress admitted to the SCBU of the Sierra Leone-China Friendship Hospital between March 2022 and March 2024. Participants were divided into two groups: 48 neonates who received nasal cannula oxygen therapy from March 2022 to March 2023 (the control group), and 45 neonates who received modified bubble CPAP therapy from April 2023 to March 2024 (the observation group). The inclusion criteria were: (1). aged 0–28 days; (2). diagnosed with respiratory distress and mouth breathing in accordance with *European Consensus Guidelines on the Management of Respiratory Distress Syndrome: 2022 Update* (10), presenting with inspiratory retractions of the suprasternal notch, bilateral supraclavicular fossae, and intercostal spaces when breathing.Exclusion criteria included any of the following: (1). absence of parental consent, (2). major congenital anomalies such as congenital heart disease which need surgery.

### Ethical consideration

The study was approved by the Institutional Review Board of Hunan Children's Hospital, China, and the Sierra Leone-China Friendship Hospital (HCHLL2020-63). The researchers elucidated the objectives, procedures, follow-up processes, and cooperation requirements of the study. Prior to enrollment, written informed consent was obtained from all participants. Data were managed anonymously and coded to maintain confidentiality. Participants were informed of their right to withdraw from the study at any time without the obligation to provide a reason.

### Intervention

All enrolled neonates received care in accordance with the study protocol that included infection control, fluid and electrolyte management, and acid-base regulation. Before the intervention, neonates’ nasopharyngeal secretions were suctioned by the nurse. All neonates underwent continuous monitoring of vital signs and respiratory status by nurses during the study.

Neonates in the control group were given routine nasal cannula oxygen therapy at 2l/min via an oxygen concentrator.

### Modified bubble CPAP therapy practice

**Figure 1:**
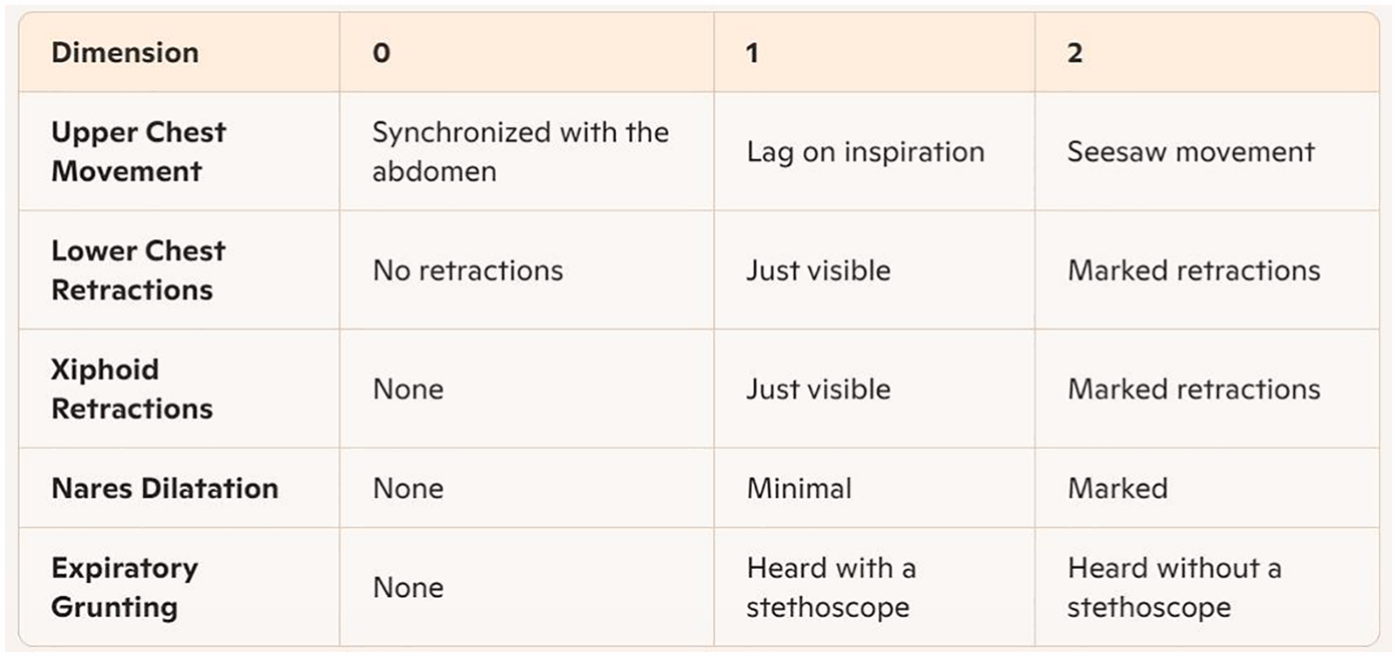
Silverman-Anderson respiratory severity score (RSS) assessment card.

Prior to applying bubble CPAP treatment in April 2023, a multidisciplinary Sierra Leone Newborn Health Focus Group was established, jointly co-led by neonatal experts from the Sierra Leone Ministry of Health and the attending from NICU of China. Comprising 1 Sierra Leonean pediatrician, 4 senior Sierra Leonean nurses, the focus group underwent specialized training on neonatal respiratory distress management and theoretical frameworks beginning in March 2023. Training emphasized clinical application of the Silverman Anderson Respiratory Severity Score (RSS) (11) to assess five clinical signs (each scored from 0 to 2), with higher scores indicating greater respiratory distress severity (Figure 1).

Teaching modalities included seminars, workshop, instructional videos, and group practice sessions. Following the initial training, the participants would receive ongoing feedback and an additional week of hands-on training. All participants passed assessments administered by the China Medical Team. The nursing staff participating in the program receive no financial compensation and their involvement was recognized by the hospital administration as part of their official duty hours.

**Figure 2:**
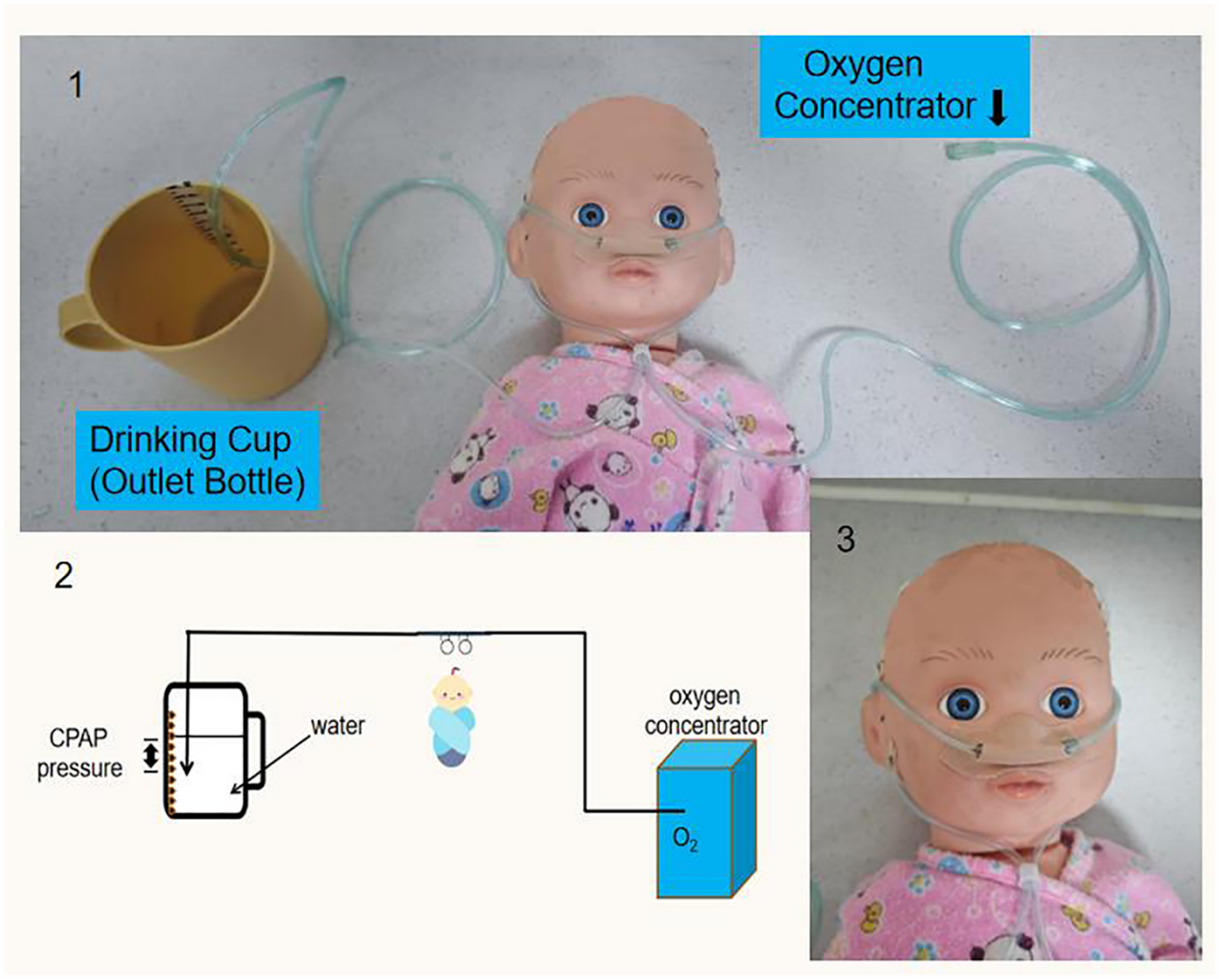
Patient undergoing modified bubble CPAP treatment.

The Sierra Leone-China Friendship Hospital SCBU relied exclusively on EverFlo 0124944 oxygen concentrators (USA-manufactured) for oxygen supply which the users could only adjust its oxygen flow, with no centralized gas source or mechanical ventilators available. To construct the modified bubble CPAP system, researchers converted a standard nasal cannula into a connecting tube using scissors, linking one end to an oxygen concentrator and the other end to an outlet bottle. Due to resource limitations in Sierra Leone, researchers further adapted the CPAP device by replacing the outlet bottle with a readily available drinking cup. The nasal prongs of the tube were then inserted into the patient's nostrils, following the standard method of nasal cannula oxygen therapy. To prevent air leakage, the researchers sealed the nasal plug with adhesive tape. An adhesive tape with a graduated scale was affixed to the inner wall of the drinking cup. This scale allowed precise measurement of the submersion depth of the outlet tube beneath the water surface, which directly dictated the Bubble CPAP pressure level generated by the device. Oxygen from the concentrator flowed through the inlet tube into the patient's airway. As the oxygen exited via the outlet tube, the pressure from the water column ensured both reduced airway resistance and sufficient pressure delivery to the nasal airway opening. The outlet bottle should be kept upright during operation to maintain positive end-expiratory pressure (PEEP) (Figure 2).

The modified bubble CPAP system was initiated at a pressure of 6 cm H_2_O, and a flow rate of 6 L/m, so the calculated oxygen concentration was 45% (12). Subsequently, the research team of China and Sierra Leone clinicians conducted assessment and monitoring, adjusting the CPAP pressure and oxygen concentration based on the neonates' clinical status while maintaining oxygen saturation between 88%–93%.

All drinking cups used in the study were brand new, undergoing surface disinfection before use. Sterile and single-use nasal cannulas compliant with all necessary safety standards were employed. Throughout the study, the trained personnel monitored the setup meticulously, with immediate replacement of any contaminated or worn components. All items were disposed after use following strict medical waste protocols to prevent cross-contamination.

### Data collection

The Respiratory Severity Score (RSS), blood oxygen saturation, heart rate (HR), and respiratory rate (RR) of the two groups were recorded upon admission and 8 h post-intervention (13, 14). A comparative analysis of clinical outcomes of the two groups was performed across these time points.

The RSS is a tool to measure the severity of respiratory failure in children with respiratory distress (15). A normal RSS is 0 point; 1–3 points indicate mild respiratory failure; 4–6 points moderate respiratory failure, and 7–10 severe respiratory failure.

Oxygen saturation was measured using a transcutaneous pulse oximeter attached to the neonate's fingertip. This device provided real-time readings of both the HR and oxygen saturation values. The RR was manually assessed by visually observing the rise and fall of the chest or abdomen; one full respiratory cycle (rise and fall) was counted as a single breath, and breaths were tallied over 60 s to calculate RR. Comparative analysis of clinical outcomes between the two groups was conducted pre- and post-intervention.

### Statistical analysis

Data processing and analysis were conducted utilizing SPSS version 25.0. Quantitative data were represented as mean ± standard deviation and evaluated using the *T*-test. Categorical data were expressed as percentages and assessed using the chi-square test. A *P*-value of less than 0.05 was regarded as indicative of statistical significance.

## Results

A total of 93 neonates were included in the study, with 48 neonates receiving nasal cannula oxygen therapy as the control group and 45 in the observation group receiving the modified bubble CPAP as their primary respiratory support. There were no significant differences between the two groups in terms of gender, age, body weight, gestational age, or mode of delivery (*P* > 0.05). Their detailed demographic characteristics are presented in Table 1.

**Table 1:** Demographic characteristics of the two groups.

Variable	Value	Observation Group (*n* = 45)	Control Group (*n* = 48)	*t/χ* ^2^	*P*
Gender	Male	27 (60%)	24 (50%)	0.313	0.576
	Female	18 (40%)	24 (50%)		
Age (hs)	Mean ± SD	26.733 ± 14.150	31.250 ± 14.942	−0.863	0.395
Body weight (kg)	Mean ± SD	3.133 ± 0.535	3.225 ± 0.586	−0.454	0.653
Gestational Age (wks)	Full-term	36 (80%)	39 (81%)	0.008	0.930
	Preterm	9 (20%)	9 (19%)		
	Mean ± SD	37.42 ± 0.232	37.71 ± 0.244	−0.927	0.354
Delivery Method	Vaginal	33 (73%)	33 (69%)	0.079	0.779
	Cesarean	12 (27%)	15 (31%)		

*P* value less than 0.05 represents statistical significance.

Upon admission, prior to initiating any interventions, the research team evaluated the RSS and measured blood oxygen saturation, heart rate, and respiratory rate in both study groups. As shown in Table 2, no statistically significant differences were observed between the groups in baseline parameters (*P* > 0.05). When comparing the five parameters of RSS, no statistically significant differences were identified between the groups before the intervention (*P* > 0.05).

**Table 2:** Comparison of parameters between two groups before intervention.

Items		Observation Group (*n* = 45)	Control Group (*n* = 48)	*t*	*P*
RSS Score	Upper chest movement	1.71 ± 0.458	1.67 ± 0.476	0.458	0.648
	Lower chest retractions	1.29 ± 0.626	1.46 ± 0.504	−1.443	0.153
	Xiphoid retractions	1.11 ± 0.532	1.08 ± 0.539	0.250	0.803
	Nares dilatation	1.53 ± 0.505	1.48 ± 0.505	0.517	0.606
	Expiratory grunting	1.76 ± 0.435	1.63 ± 0.489	1.357	0.178
	Total Score	7.40 ± 0.986	7.31 ± 0.793	0.273	0.787
Oxygen saturation		73.60 ± 10.636	77.25 ± 4.359	−1.265	0.216
Heart rate (beats/min)		153.13 ± 5.998	154.69 ± 3.807	−0.867	0.393
Respiratory rate (times/min)		47.87 ± 4.103	50.19 ± 4.020	−1.59	0.123

Due to resource limitations, direct monitoring of the oxygen concentration was not viable. However, based on the oxygen flow rate, the calculated oxygen concentration received by the neonates was approximately 29%. After 8 h of intervention, the observation group demonstrated significantly lower RSS, higher blood oxygen saturation, and reduced respiratory rates compared to controls. All RSS parameters scores dropped (*P* < 0.05), but no significant differences in heart rate were observed between groups (*P* > 0.05) (see Table 3).

**Table 3:** Comparison of parameters between two groups after intervention.

Items		Observation Group (*n* = 45)	Control Group (*n* = 48)	*t*	*P*
RSS Score	Upper chest movement	1.56 ± 0.503	1.75 ± 0.438	−1.993	0.049
	Lower chest retractions	1.00 ± 0.522	1.56 ± 0.501	−5.299	<0.001
	Xiphoid retractions	0.67 ± 0.477	1.00 ± 0.546	−3.128	0.002
	Nares dilatation	0.96 ± 0.520	1.44 ± 0.542	−4.369	<0.001
	Expiratory grunting	1.09 ± 0.557	1.71 ± 0.459	−5.867	<0.001
	Total Score	5.33 ± 1.447	7.50 ± 1.095	−4.719	<0.001
Oxygen saturation		91.07 ± 8.940	73.00 ± 8.809	5.666	<0.001
Heart rate (beats/min)		141.60 ± 8.830	145.06 ± 22.161	−5.564	0.577
Respiratory rate (times/min)		43.47 ± 3.833	53.19 ± 5.357	−5.775	<0.001

A within-group comparative analysis of pre- and post-intervention parameters demonstrated that the control group showed no statistically significant changes in total RSS scores, oxygen saturation, heart rate, or respiratory rate (*P* > 0.05). However, expiratory grunting scores increased significantly (*P* < 0.05) as shown in Table 4.

**Table 4:** Comparison of data before and after intervention in the control group.

Items		Before intervention	After intervention	*t*	*P*
RSS Score	Upper chest movement	1.67 ± 0.476	1.75 ± 0.438	−1.663	0.103
	Lower chest retractions	1.46 ± 0.504	1.56 ± 0.501	−1.944	0.058
	Xiphoid retractions	1.08 ± 0.539	1.00 ± 0.546	1.663	0.103
	Nares dilatation	1.48 ± 0.505	1.44 ± 0.542	0.573	0.569
	Expiratory grunting	1.63 ± 0.489	1.71 ± 0.459	−2.067	0.044
	Total Score	7.31 ± 0.793	7.50 ± 1.095	−0.555	0.583
Oxygen saturation		77.25 ± 4.359	73.00 ± 8.809	1.730	0.098
Heart rate (beats/min)		154.69 ± 3.807	145.06 ± 22.161	−1.861	0.067
Respiratory rate (times/min)		50.19 ± 4.020	53.19 ± 5.357	−1.792	0.083

Post-intervention analysis revealed that the observation group exhibited statistically significant improvements compared to pre-intervention baseline: lower RSS scores, heart rate and respiratory frequency, and higher oxygen saturation (*P* < 0.01), as shown in Table 5.

**Table 5:** Comparison of data before and after intervention in the observation group.

Items		Before intervention	After intervention	*t*	*P*
RSS Score	Upper chest movement	1.71 ± 0.458	1.56 ± 0.503	2.461	0.018
	Lower chest retractions	1.29 ± 0.626	1.00 ± 0.522	3.833	<0.001
	Xiphoid retractions	1.11 ± 0.532	0.67 ± 0.477	4.781	<0.001
	Nares dilatation	1.53 ± 0.505	0.96 ± 0.520	6.240	<0.001
	Expiratory grunting	1.76 ± 0.435	1.09 ± 0.557	6.992	<0.001
	Total Score	7.40 ± 0.986	5.33 ± 1.447	4.571	<0.001
Oxygen saturation		73.60 ± 10.636	91.07 ± 8.940	−4.869	<0.001
Heart rate (beats/min)		153.13 ± 5.998	141.60 ± 8.830	4.184	<0.001
Respiratory rate (times/min)		47.87 ± 4.103	43.47 ± 3.833	3.035	0.005

After treatment, 18 out of 48 neonates in the control group died, and 5 of 45 neonates in the observation group died. Due to the limited medical conditions in Sierra Leone, autopsies were not performed on all the death cases. Although respiratory failure might be one of the causes of deaths in these cases, the exact cause of death was still unclear. Consequently, 88% (40/45) of the neonates treated with modified bubble CPAP survived to discharge. In contrast, the survival rate for neonates diagnosed with RDS who received a nasal cannula for respiratory support was 62.5% (30/48). Statistical analysis showed that the survival rate of the observation group was significantly higher than that of controls (*χ*^2^ = 8.689, *P* = 0.003).

## Discussion

Bubble CPAP has emerged as a vital intervention for neonatal respiratory distress in low- and middle-income countries, with expanding adoption for its cost-effectiveness, ease of use, and proven clinical efficacy. Studies have shown its capacity to reduce reliance on mechanical ventilation—a technology often scarce in low-resource settings. The system's operational simplicity, convenience, and effectiveness (16) enable rapid competency development of healthcare workers by training, making it a practical solution in settings with limited specialized staff. Despite these benefits, extensive further evidence-based research is required to optimize its implementation and ensure consistent outcomes across diverse healthcare environments (17).

This study introduces an innovative modification of bubble CPAP device to address the resource constraints, especially in emergency situations where conventional medical supplies may be unavailable. By leveraging readily accessible materials and a streamlined design, the modified bubble CPAP device ensures reliable delivery of respiratory support even in resource-constrained environments. This approach not only enhances the accessibility of life-saving technology, but also underscores the critical role of adaptive innovation in healthcare. Widespread adoption of this approach could potentially save numerous lives by offering a practical and cost-efficient solution to respiratory distress in underserved regions.

In our study, the demographic parameters (gender, age, body weight, gestational age, and delivery mode) showed no significant intergroup differences, confirming effective randomization and minimizing confounding bias. The study's design ensured that observed post-intervention differences can be attributed to the intervention itself rather than pre-existing conditions.

After 8 h of therapy, the observation group demonstrated significantly better outcomes in RSS, blood oxygen saturation, and respiratory rate compared to controls. The lower RSS score indicated that less respiratory distress, attributable to the continuous positive airway pressure provided by modified bubble CPAP. This positive pressure stabilized the alveoli patency during both inhalation and exhalation, reducing the work of breathing, enhancing gas exchange efficiency, and higher blood oxygen saturation levels (18). The observation group's lower respiratory rate reflected improved breathing efficiency as a direct benefit of the positive airway pressure provided by bubble CPAP (19). These findings suggested that modified bubble CPAP surpasses nasal cannula oxygen therapy in stabilizing and improving respiratory function in neonates with RDS.

The study also performed within-groups analysis on indicators before and after the intervention to assess the theraputic effects. No significant pre-post differences in the control group for RSS, oxygen saturation, heart rate, or respiratory rate were found, suggesting that nasal cannula oxygen therapy did not significantly improve the neonates' respiratory parameters. However, the scores of expiratory grunting, a compensatory mechanism produced by neonates to maintain functional residual capacity and improve oxygenation (20) increased significantly after intervention, indicating the neonates' persistent respiratory distress despite oxygen therapy. This finding suggests that nasal cannula oxygen therapy may not have been effective in fully addressing the respiratory needs of these neonates, thereby leading to a compensatory rise in expiratory grunting.

In contrast, the observation group showed statistically significant decreases in RSS and respiratory rate, as well as an increase in oxygen saturation compared to those before the intervention (*P* < 0.01). These improvements confirmed the dual advantages of modified bubble CPAP therapy: (1) Improving oxygenation while reducing work of breathing. The continuous positive pressure it generated helped maintain a higher functional residual capacity (FRC)—the post-exhalation lung volume- which optimized oxygenation efficiency and lowered the need for higher oxygen concentrations (21). (2) Preventing atelectasis (collapse of part of or the entire lung), a common issue in neonates with respiratory distress. By keeping the alveoli patency, bubble CPAP strengthened lung compliance and gas exchange (22, 23). Additionally, none significant inter-group differences in heart rate (*P* > 0.05) suggested that such therapy did not impose additional cardiovascular stress on neonates, indicating that the improved respiratory parameters were achieved without compromising cardiovascular stability (24).

The observation group had a higher survival rate (88%) than the control group (62.5%), which was likely due to the less effective respiratory support from nasal cannula oxygen therapy. Despite limitations in autopsy availability precluded the analysis for exact causes of death, respiratory distress in full-term newborns might be speculated as a probable contributor. In resource-constrained settings with limited diagnostic and treatment capabilities, this survival disparity underscores modified bubble CPAP's superiority in improving outcomes of neonates with RDS through better respiratory support (25).

The cost-effectiveness and user-friendly design of modified bubble CPAP make it a particularly viable option for neonatal care in low- and middle-income countries, where advanced respiratory support may be inaccessible (26). More research and policy support are essential to fully realize its advantages and ensure that more neonates can receive the respiratory support they need.

## Limitation

Although the findings are promising, it is important to recognize several limitations inherent in this study. The relatively small sample size (*n* = 93 neonates) may compromise the robustness and generalizability of the results, highlighting the need for validation through studies conducted on a larger scale. Additionally, the single-center design may limit the applicability of the findings across diverse settings, particularly in regions with varying standards of neonatal care. To address these limitations, it is crucial to conduct large-scale, multi-center trials employing advanced methodologies to validate and optimize the use of modified bubble CPAP therapy in resource-limited environments. Such efforts will also facilitate its integration into routine neonatal care, thereby maximizing its potential benefits.

## Conclusion

The study demonstrated that modified bubble CPAP therapy improved respiratory outcomes and survival rates in neonates with respiratory distress in a resource-limited setting in rural Sierra Leone. The implementation of this low-cost, simplified CPAP system holds significant potential for scaling neonatal respiratory care and to improve survival rates in low- and middle-income countries.

## Data Availability

The raw data supporting the conclusions of this article will be made available by the authors, without undue reservation.

## References

[1] Lawn JE Blencowe H Waiswa P Amouzou A Mathers C Hogan D Stillbirths: rates, risk factors, and acceleration towards 2030. Lancet. (2016) 6736(15):00837–5. 10.1016/S0140 26794078

[2] World Health Organization. WHO Methods and Data Sources for Country-level Causes of Death 2000-2016, Department of Information, Evidence and Research. Geneva: WHO (2018).

[3] Bulimba M Cosmas J Abdallah Y Massawe A Manji K. Early outcomes of preterm neonates with respiratory distress syndrome admitted at muhimbili national hospital, a prospective study. BMC Pediatr. (2022) 22(1):731. 10.1186/s12887-022-03731-2 36550480 PMC9773513

[4] Lista G Castoldi F Cavigioli F Bianchi S Fontana P. Alveolar recruitment in the delivery room. J Matern Fetal Neonatal Med. (2012) 25(1):39–40. 10.3109/14767058.2012.663164 22313342

[5] WHO Department of Maternal, Newborn, Child and Adolescent Health, WHO Department of Reproductive Health and Research. WHO recommendations on interventions to improve preterm birth outcomes: highlights and key messages from the world health organization's 2015 global recommendations. World Health Organization (2015). Available at: https://iris.who.int/handle/10665/183055 (Accessed April 28, 2023).

[6] Ramaswamy VV Abiramalatha T Bandyopadhyay T Shaik NB Pullattayil SAK Cavallin F Delivery room CPAP in improving outcomes of preterm neonates in low-and middle-income countries: a systematic review and network meta-analysis. Resuscitation. (2022) 170:250–63. 10.1016/j.resuscitation.2021.10.027 34757058

[7] Ho JJ Subramaniam P Davis PG. Continuous positive airway pressure (CPAP) for respiratory distress in preterm infants. Cochrane Database Syst Rev. (2020) 10(10):CD002271. 10.1002/14651858.CD002271.pub3 33058208 PMC8094155

[8] Owen LS Manley BJ Davis PG Doyle LW. The evolution of modern respiratory care for preterm infants. Lancet. (2017) 389(10079):1649–59. 10.1016/S0140-6736(17)30312-4 28443559

[9] McAdams RM Hedstrom AB DiBlasi RM Mant JE Nyonyintono J Otai CD Implementation of bubble CPAP in a rural Ugandan neonatal ICU. Respir Care. (2015) 60(3):437–45. 10.4187/respcare.03438 25389349

[10] Sweet DG Carnielli VP Greisen G Hallman M Klebermass-Schrehof K Ozek E European consensus guidelines on the management of respiratory distress syndrome: 2022 update. Neonatology. (2023) 120(1):3–23. 10.1159/000528914 36863329 PMC10064400

[11] Switchenko N Kibaru E Tsimbiri P Grubb P Anderson Berry A Fassl B. Implementation of a bubble CPAP treatment program for sick newborns in Nakuru, Kenya: a quality improvement initiative. Glob Pediatr Health. (2020) 7:1–10. 10.1177/2333794X20939756 32821774 PMC7412892

[12] Al-Lawama M Alkhatib H Wakileh Z Elqaisi R AlMassad G Badran E Bubble CPAP therapy for neonatal respiratory distress in level III neonatal unit in Amman, Jordan: a prospective observational study. Int J Gen Med. (2018) 12:25–30. 10.2147/IJGM.S185264 30636889 PMC6307683

[13] Roehr CC. CPAP in neonates: current methods and further improvements. In: Esquinas AM, editor. Noninvasive Ventilation in Sleep Medicine and Pulmonary Critical Care: Critical Analysis of 2018–19 Clinical Trials. Cham: Springer (2020). p. 465–75.

[14] Keerthan RM Pudi N Greeshma G Kavita K Sharma J. A systematic review, meta-analysis and economic evaluation on Neonatal cpap. Comput Math Biophys. (2022) 10(1):68–86. 10.1515/cmb-2022-0133

[15] Cavallin F Balestri E Calia M Biasci F Tolera J Pietravalle A Training on the Silverman and Andersen score improved how special care unit nurses assessed neonatal respiratory distress in a low-resource setting. Acta Paediatr. (2022 Oct) 111(10):1866–9. 10.1111/apa.16450 35700104

[16] Martin S Duke T Davis P. Efficacy and safety of bubble CPAP in neonatal care in low and middle income countries: a systematic review. Arch Dis Child Fetal Neonatal Ed. (2014) 99(6):495–504. 10.1136/archdischild-2013-305519 25085942

[17] Mcadams RM. Bubble CPAP may be safe and efficacious for neonates in low and middle income countries, but more evidence is needed. Evid Based Med. (2015) 20(2):62. 10.1136/ebmed-2014-110135 25666020

[18] Baldursdottir S Falk M Donaldsson S Jonsson B Drevhammar T. Basic principles of neonatal bubble CPAP: effects on CPAP delivery and imposed work of breathing when altering the original design. Arch Dis Child Fetal Neonatal Ed. (2020) 105(5):550–4. 10.1136/archdischild-2019-318073 32047029 PMC7547905

[19] Bharadwaj SK Alonazi A Banfield L Dutta S Mukerji A. Bubble versus other continuous positive airway pressure forms: a systematic review and meta-analysis. Arch Dis Child Fetal Neonatal Ed. (2020) 105(5):526–31. 10.1136/archdischild-2019-318165 31969457

[20] Neumann RP von Ungern-Sternberg BS. The neonatal lung–physiology and ventilation. Paediatr Anaesth. (2014) 24(1):10–21. 10.1111/pan.12280 24152199

[21] Aly H Mohamed MA. An experience with a bubble CPAP bundle: is chronic lung disease preventable? Pediatr Res. (2020) 88(3):444–50. 10.1038/s41390-020-0763-3 31952073 PMC7223768

[22] Singh R Munian LP Memela NA. Management of neonates with respiratory distress syndrome in resource-limited settings. S Afr Fam Pract (2004). (2024) 66(1):e1–7. 10.4102/safp.v66i1.5938 38832392 PMC11151355

[23] Sabry AM Bastawy RS Abdullatif DAK Edris AAF El-Baz MS Clinical predictors for outcome of continuous positive airway pressure in respiratory distress syndrome in preterms: single center study. Pediatr Sci J. (2023) 3(1):1–11. 10.21608/cupsj.2022.140797.1053

[24] Anne RP Murki S. Noninvasive respiratory support in neonates: a review of current evidence and practices. Indian J Pediatr. (2021) 88(7):670–8. 10.1007/s12098-021-03755-z 34075532 PMC8169393

[25] Vc LK Patla VKR Vadije PR Murki S Subramanian S Injeti G Assessing the diagnostic accuracy of lung ultrasound in determining invasive ventilation needs in neonates on non-invasive ventilation: an observational study from a tertiary NICU in India. Eur J Pediatr. (2024) 183(2):939–46. 10.1007/s00431-023-05356-8 38052734

[26] Bailes SA Firestone KS Dunn DK McNinch NL Brown MF Volsko TA. Evaluating the effect of flow and interface type on pressures delivered with bubble CPAP in a simulated model. Respir Care. (2015) 61(3):333–9. 10.4187/respcare.04251 26534997

